# Radiographic and functional outcomes of shelf acetabuloplasty versus conservative management in legg-calvé-perthes disease: an age- and gender-matched study including healthy controls for isokinetic hip muscle strength

**DOI:** 10.1007/s00264-025-06588-z

**Published:** 2025-06-25

**Authors:** Mehmet Demirel, İlhan Sulejmani, Yaşar Gökçeoğlu, Türker Şahinkaya, Yavuz Sağlam, Fuat Bilgili

**Affiliations:** 1 İstanbul University, Faculty of Medicine, Department of Orthopedics and Traumatology, İstanbul, Türkiye; 2 İstanbul Üniversitesi, Faculty of Medicine, Department of Sport Medicine, İstanbul, Türkiye

**Keywords:** Legg-calvé-perthes disease, Shelf acetabuloplasty, Conservative treatment, Isokinetic muscle strength, Salvage procedure, Radiographic outcomes

## Abstract

**Introduction:**

Shelf acetabuloplasty, one of surgical containment methods, have been employed to preserve hip joint congruity in the management of Legg-Calvé-Perthes disease (LCPD). However, its long-term effect on radiographic and functional outcomes remains unclear due to limited evidence. Moreover, comparative studies against conservative treatment are lacking. This study aimed to (1) compare the mid- to long-term outcomes between children with advanced-stage LCPD treated with shelf acetabuloplasty and those receiving conservative management, and (2) evaluate isokinetic hip muscle strength compared to age- and gender-matched healthy controls.

**Materials and methods:**

This retrospective age- and gender-matched study included 28 children with unilateral LCPD, divided into Shelf (*n* = 14) and Conservative (*n* = 14) treatment groups. A healthy control group (*n* = 14) was also recruited for isokinetic comparisons. Radiographic outcomes were assessed using modified Stulberg classification and several quantitative parameters. Functional outcomes were assessed using the Harris Hip Score (HHS) and isokinetic testing of hip muscle strength.

**Results:**

The Shelf group (median follow-up: 5.5 years, IQR: 4–7) showed significantly better HHS (67.9 ± 15.9) compared to the Conservative group (median follow-up: 6 years, IQR: 5–8) (54.6 ± 13.3; *p* = 0.024) at the final follow-up. Shelf acetabuloplasty also resulted in significantly improved radiographic parameters, including centre–edge angle (*p* < 0.001) and femoral head coverage (*p* = 0.002). Isokinetic testing revealed that the Conservative group had significantly lower hip extension (*p* = 0.021), abduction (*p* = 0.018), and adduction (*p* = 0.027) torque values, as well as greater muscle fatigue (*p* = 0.014). In contrast, the Shelf and Control groups exhibited comparable performance in most strength and endurance parameters.

**Conclusions:**

Shelf acetabuloplasty, when applied as a salvage procedure in advanced-stage LCPD, may provide better functional outcomes and improved hip muscle performance compared to conservative treatment, despite comparable long-term femoral head morphology. Following Shelf acetabuloplasty, comparable hip flexor and extensor strength to healthy controls can be expected, although mild abductor and adductor weakness may persist.

## Introduction

Legg-Calvé-Perthes disease (LCPD), characterized by avascular necrosis of the capital femoral epiphysis, is a self-limiting developmental disorder of the hip in children. If not properly managed, this condition may lead to femoral head deformity, impaired joint function, and long-term sequelae, such as osteoarthritis [1]. Although the exact aetiology remains poorly understood, it is widely accepted that both biological and mechanical factors contribute to its pathogenesis [2]. During the disease progression, LCPD follows four well-established stages, as defined by the Waldenström classification: initial, fragmentation, reossification, and healed stages [3].

Given the complex etiology of LCPD, developing effective management strategies is crucial for preventing long-term complications. Nonetheless, treatment approaches remain controversial because of varying outcomes following different therapeutic methods [1, 2]. The primary goal of LCPD management is to prevent femoral head deformity and maintain hip joint congruity through therapeutic approaches tailored to the patient’s age, disease severity, and stage. Containment therapy, which maintains the femoral head properly within the acetabulum throughout the disease course, is regarded the cornerstone of LCPD management. This approach, which can be performed either surgically or conservatively, facilitates the remodeling of the femoral head while minimizing deformity [4]. Among surgical containment methods, *shelf acetabuloplasty* aims to maintain the femoral head within the acetabulum by adding a new bone surface to the acetabular roof, thereby facilitating remodeling in non-load-bearing areas [5]. Initially, this technique was primarily utilized as a salvage procedure to prevent deformities in the advanced stages of the disease [6, 7]. However, in recent years, it has also been applied during the early stages to promote femoral head remodeling and preserve hip joint congruity [8].

Shelf acetabuloplasty has been reported to effectively remodel the femoral head and preserve hip joint congruity in LCPD, yielding favourable clinical and radiograpgical outcomes.[4, 6–8]. However, its long-term impacts on functional outcomes and its ability to delay complications such as osteoarthritis remain elusive due to limited high-quality evidence from controlled studies [9]. Moreover, while the functional outcomes and muscle strength of children treated with this method are of significant interest, studies addressing these specific aspects are sparse. Additionally, to our knowledge, the lack of controlled research directly comparing the results of shelf acetabuloplasty with conservative management in patients with similar disease severity hinders the ability to reach solid conclusions about the efficacy of these approaches.

The primary objectives of this study were to (1) compare the mid- and long-term clinical and radiographic outcomes of age- and gender-matched children with advanced-stage LCPD treated with shelf acetabuloplasty versus conservative treatment and (2) investigate differences in isokinetic hip muscle strength between treated children and age- and gender-matched healthy volunteers. This study hypothesizes that shelf acetabuloplasty provides clinically and functionally superior outcomes compared with conservative treatment in children with LCPD when applied as a salvage procedure.

## Patients and methods

### Study design and participants

This retrospective study was conducted in the Department of Orthopaedics and Traumatology at a single university hospital between 2010 and 2021. The inclusion criteria for the study were: I) a diagnosis of unilateral LCPD, II) classified as Herring lateral column group B, B/C, or C, III) treated either surgically with shelf acetabuloplasty or conservatively (e.g., physiotherapy, crutches), IV) a minimum of three years of follow-up, V) presence of at least one ‘’head at risk’’ sign on initial radiographs, VI) age at diagnosis of ≥ six years, VII) complete clinical and radiographic records, and VIII) willingness to participate in the study. The exclusion criteria were: I) a history of metabolic, systemic, or rheumatological diseases, II) lost to follow-up, III) incomplete medical records, IV) previous history of surgery for LCPD, and V) refusal to participate in the study.

A total of 38 children diagnosed with LCPD were initially evaluated. After excluding two participants (one due to lost to follow-up and one had incomplete clinical or radiographic data), the final study population consisted of 36 children. Of these, 19 children were assigned to the *Conservative Group*, and 17 children undergoing shelf acetabuloplasty formed *the Shelf Group*.

After identifying eligible participants for both groups, age- and gender-matching was performed to ensure comparability, particularly for isokinetic muscle strength assessment. Age-matching was conducted within an acceptable range of ± one year. The matching process was carried out in two stages. First, patients in the Shelf (*n* = 17) and Conservative (*n* = 19) groups were matched in a 1:1 ratio based on age (± 1 year) and gender. Subsequently, three patients from the Shelf group and five patients from the Conservative group could not be matched and were excluded from further analysis. Control group participants were then selected using the same matching criteria (± 1 year age difference and identical gender distribution) to correspond with the previously paired Shelf and Conservative patients. The control group consisted of volunteers from the local hospital community, all of whom had no identified orthopedic abnormalities that could influence muscle strength or hip function.

After the matching process, the final cohort comprised 14 patients in the Conservative Group, 14 patients in the Shelf Group, and 14 healthy participants in the Control Group. Parents were informed that medical records could only be used for scientific purposes, and written informed consent was obtained. The study protocol was approved by our institutional ethics committee and conducted in accordance with the Declaration of Helsinki guidelines.

### Radiographic evaluation

Radiographic evaluations were performed at baseline and during follow-up. On admission, all children with LCPD were examined using the Waldenström classification to identify the disease stage [3]. The Herring Lateral Pillar Classification was determined on initial radiographs to identify the severity of femoral head involvement in both treatment groups [10]. The modified Stulberg classification was utilized to investigate radiographic outcomes at the final follow-up, and then hips were categorized into three groups: good (spherical femoral head, Stulberg I–II), fair (ovoid femoral head, Stulberg III), and poor (flat femoral head, Stulberg IV–V) [11].

Poor prognostic radiographic findings, also known as"head at risk"signs, were examined on initial radiographs, including *lateral subluxation*, *Gage sign*, *calcification in the lateral column*, *metaphyseal changes*, *horizontal growth plate* [12].

**Fig. 1:**
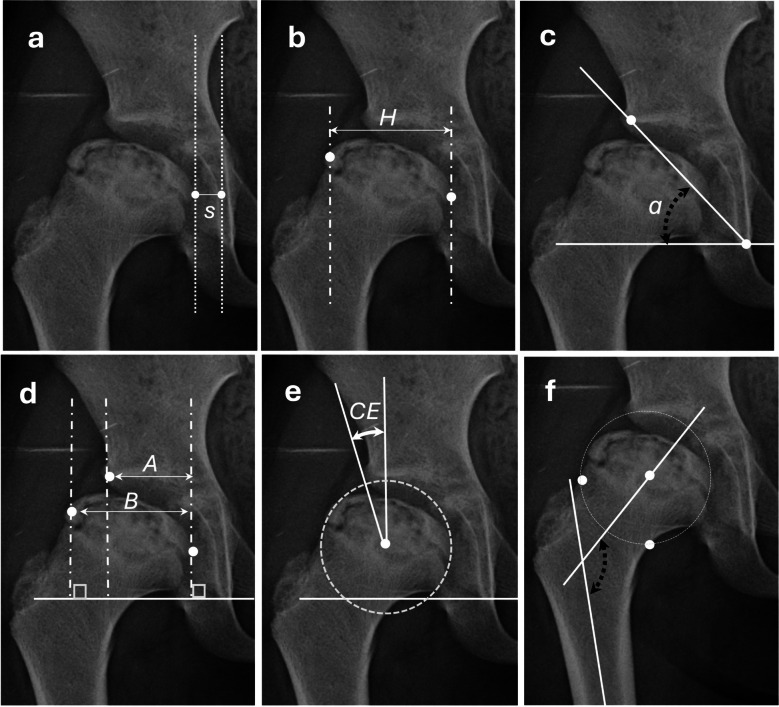
Representation of quantitative radiographic measurements (**a**) Femoral head subluxation ratio = S affectedside/S unaffectedside, **b** Femoral head size ratio = H affectedside/H unaffectedside, **c** Sharp angle (α), **d** Femoral head coverage ratio = A/B × 100, **e** Wiberg center–edge angle (CE), **f** Femoral neck-shaft angle (NSα)

On the initial and final follow-up radiographs, the outcomes of conservative treatment and the shelf acetabuloplasty procedure were assessed using various quantitative radiographic measurements, including *the femoral head subluxation ratio*, *femoral head size ratio*, *Sharp angle*, *femoral head coverage ratio*, *Wiberg centre–edge angle*, and *neck-shaft angle *[6, 7]. The femoral head subluxation ratio was calculated as the width of the medial joint space, expressed as a ratio relative to the unaffected hip [7]. These measurements are demonstrated in Fig. 1. Possible complications associated with the shelf procedure, such as graft resorption, proximal migration, and growth disturbances of the lateral acetabulum, were systematically examined and documented on follow-up radiographs.

Radiographic assessments were conducted exclusively on children treated for LCPD. Radiographs were reviewed independently by two experienced pediatric orthopaedic surgeons blinded to the treatment groups to minimize bias.

### Clinical evaluation

Clinical outcomes were assessed using the following methods. Harris Hip Score (HHS) [13] was assessed exclusively in children treated for LCPD at the final follow-up to measure hip function and mobility.

Isokinetic Muscle Strength measurements were performed at the final follow-up based on a standardized isokinetic testing protocol using the CYBEX 350 isokinetic dynamometer (CYBEX HUMAC, version 2009; Computer Sports Medicine Inc, Stoughton, MA) outlined below. All tests were supervised by an experienced sports medicine specialist to ensure proper execution and reliability of the results. Measurements were performed for hip flexion, extension, adduction, and abduction.

For each tested movement, the following parameters were recorded:

- PT (Peak Torque) = maximum torque generated during the test (Newton-meters, Nm);
- WfR (Work Fatigue Ratio): The ratio of work performed across the test (percentage, %);

Testing procedures were conducted individually, with participants performing a standardized warm-up on a cycle ergometer at 25 Watts for two min prior to testing. For hip abduction and adduction, a standardized side-lying position was used with a backrest to minimize compensatory movements [14]. Testing began with the dominant leg, followed by the non-dominant leg at an angular velocity of 60°, as this seems to represent velocity used during activities of daily life [15, 16]. Lower (e.g., 30°/s) and higher velocities (e.g., 180°/s) were avoided to ensure consistency and practicality [15]. Before the actual testing, participants performed three submaximal and one maximal repetition to familiarize themselves with the protocol. During testing, three consecutive maximal repetitions were completed for each muscle group without pauses between repetitions. A two min rest period was provided between tests for different muscle groups. Standardized verbal encouragement was given to ensure maximum effort, and feedback was continuously provided regarding the number of repetitions remaining [15].

Data from the three consecutive maximal contractions were recorded and used for statistical analyses. Measurements were taken separately for the affected hip and the unaffected hip for the Shelf and Conservative Groups. Conversely, the mean values of both hips were used for Control group as a reference.

### Conservative treatment protocol

Our clinic implemented a tailored conservative treatment for LCPD, focusing on alleviating symptoms and preserving hip joint mobility. Physiotherapy was essential in employing exercises to preserve joint range of motion and augment periarticular muscle strength, especially for hip abduction and rotation movements. High-impact activities such as jumping and running were restricted, yet swimming and bicycling were recommended. Gait aids such as crutches were utilized during early LCPD stages. Non-steroidal anti-inflammatory medicines were given to alleviate inflammation and discomfort, supplemented with adjuvant treatments such as heat or ice applications.

### Indications for shelf acetabuloplasty

At our institution, the primary indication for shelf acetabuloplasty as a salvage procedure was any degree of epiphyseal extrusion without hinge abduction in children older than seven years of age during the late fragmentation or reossification stages of the disease.

### Operative technique

The shelf procedure was performed following the Staheli technique [17] via a bikini skin incision. The gluteus medius and tensor fascia lata were lifted from the outer table of the ilium, while the reflected head of the rectus femoris was detached, mobilized, and carefully preserved. Corticocancellous bone grafts were harvested from the iliac wing and inserted into the prepared slot to cover the femoral head laterally and anteriorly. The reflected head of the rectus femoris was reattached over the graft, and additional cancellous bone grafts were packed above the shelf. None of the patients received psoas release. If required, adductor release was performed through a seperate incision during the procedure.

Postoperatively, all children were maintained non-weight-bearing for six weeks in a single-leg spica cast. They were then encouraged to mobilize their hips and gradually bear weight. Postoperative rehabilitation included preserving range of motion and strengthening muscles around the hip joint.

### Statistical analysis

All statistical analyses were conducted utilizing SPSS version 28.0 (IBM Corp., Armonk, New York, USA). The Shapiro–Wilk test was employed to evaluate the normality of the data. For comparisons between two groups, the Student's t-test was utilized for regularly distributed data, while the Mann–Whitney U test was applied for non-normally distributed data. Categorical variables were analyzed using the Chi-square test. For comparisons among three groups, the ANOVA test was used for normally distributed data, and the Kruskal–Wallis test was applied for non-normally distributed data. A p-value of < 0.05 was considered statistically significant. Interobserver reliability for quantative radiographic measurements was assessed by calculating the intraclass correlation coefficient (ICC). The agreement was considered excellent for ICC > 0.80, very good for 0.70 to 0.80, good for 0.60 to 0.70, fair for 0.40 to 0.60, and poor for < 0.40. For other nominal radiographic parameters, interobserver reliability was evaluated using Cohen’s kappa (κ) coefficient, with agreement categorized as perfect (κ > 0.80), substantial (0.61–0.80), moderate (0.41–0.60), fair (0.21–0.40), and slight (< 0.20).

## Results

### Baseline characteristics

A total of 28 children with unilateral LCPD were included, with 14 patients each in the Shelf and Conservative groups. The median age at diagnosis was significantly higher in the Shelf group (10 years, IQR: 8–11) compared to the Conservative group (8 years, IQR: 7–9) (*p* = 0.004). The median follow-up duration was similar between groups (Shelf: 5.5 years, Conservative: 6 years; *p* = 0.07). Gender distribution and age at final follow-up were comparable between the groups (Table 1).

**Table 1:** Demographic characteristics of patients in the Shelf Acetabuloplasty and Conservative Treatment groups

Variables	Conservative Group	Shelf Group	*P* values*	Control Group
Gender *(n), boys/girls*	13 boys, 1 girl	13 boys, 1 girl	1.000^a^	13 boys, 1 girl
Age at the time of diagnosis (year),*median [IQR] (mean, min–max)*	8 [7–9] (7.79, 6–11)	10 [8-11] (9.79, 6–12)	*0.004*	
Age at surgery (year), *median [IQR] (mean, min–max)*	—	11 [8-12] (10, 6–13)		
Age at final follow-up (year), *median [IQR] (mean, min–max)*	15 [14-16] (15, 13–17)	15 [14-16] (15.21, 13–17)	0.527^b^	15 [14-16] (15, 13–17)
Follow-up duration (year), *median [IQR] (mean, min–max)*	6 [5-8] (6.79, 4–10)	5.50 [4-7] (5.50, 3–8)	0.07^b^	

A statistically significant difference was considered at *p* < 0.05

^a^Chi-square test; ^b^Mann-Whitney U test

^*^P values represent the statistical comparison between the Conservative Group and the Shelf Group

### Radiographic outcomes

Interobserver agreement was excellent for quantitative radiographic measurements, with ICC values ranging from 0.80 to 0.99. For nominal radiographic parameters, interobserver agreement was classified as perfect, with kappa values ranging from 0.81 to 0.98.

#### Radiographic staging and classifications

**Fig. 2:**
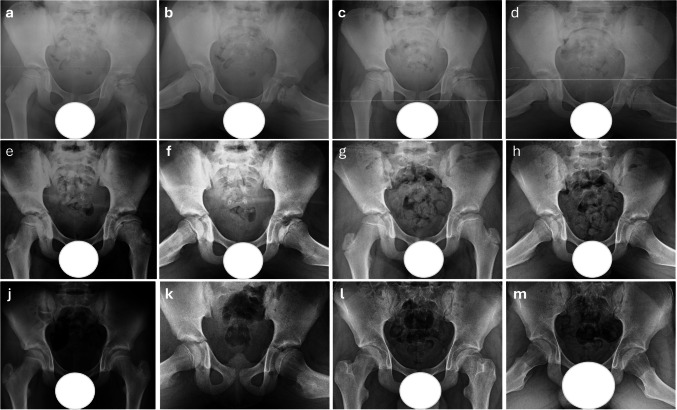
Serial radiographic follow-up of an 8-year-old male patient with Legg-Calvé-Perthes disease treated conservatively. Initial anteroposterior (AP) and frog-leg lateral hip radiographs at the time of diagnosis (8 years old), demonstrating Waldenström stage 3 (early reossification stage) with lateral subluxation and multiple"head at risk"signs, including metaphyseal cysts, Gage sign, and calcification in the lateral column (**a**, **b**). Radiographs at the 1-year follow-up, showing ongoing reossification with partial femoral head remodeling, though subluxation persists (**c**, **d**). Radiographs at the 3-year follow-up, indicating remodeling phase, with continued femoral head reshaping and improved acetabular containment. However, residual deformity remains evident (**e**, **f**). Radiographs at the 5-year follow-up, demonstrating remodeling phase with further improvement in femoral head sphericity and acetabular congruence (**g**, **h**). Radiographs at the 6-year follow-up, showing excellent femoral head remodeling, with near-complete restoration of sphericity and improved hip joint congruency (**j**, **k**). Radiographs at the 8-year follow-up, demonstrating complete remodeling, with the resolution of lateral subluxation. The femoral head has achieved *Stulberg Class I* morphology, with a subluxation ratio of 1, indicating full containment and joint congruency (**l**, **m**)

**Fig. 3:**
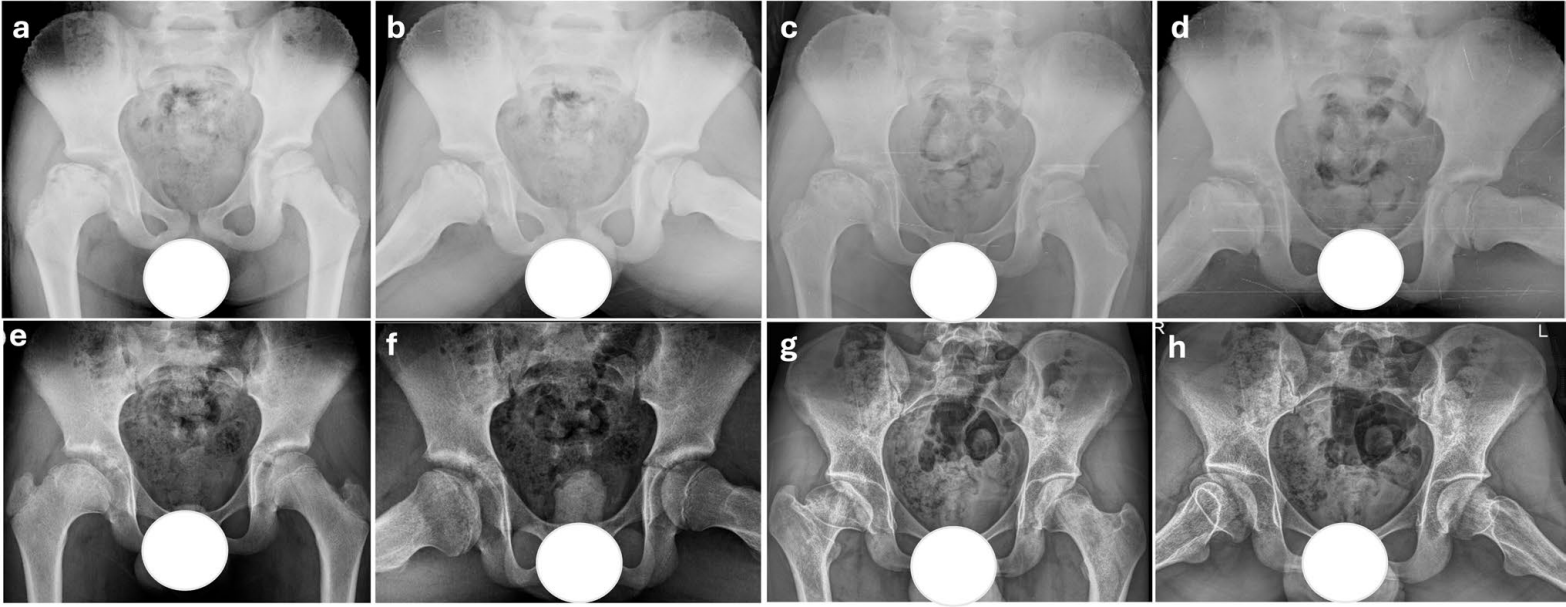
Serial Radiographic Follow-up of a 9-Year-Old Patient with LCPD Treated Conservatively. Initial radiographs at the fragmentation stage demonstrate complete collapse of the lateral pillar, classifying the patient as Herring Group C. Lateral subluxation of the femoral head is evident, accompanied by marked epiphyseal extrusion (**a**, **b**). 1-year follow-up: Early remodeling changes are observed. While femoral head sphericity is slightly altered, it remains largely preserved (**c**, **d**). 3-year follow-up: Progressive remodeling continues; however, flattening of the acetabulum becomes apparent, along with increasing loss of femoral head sphericity (**e**, **f**). 7-year follow-up: Final follow-up radiographs demonstrate poor outcomes with a Stulberg Class V deformity, indicating severe femoral head flattening and joint incongruity (**g**, **h**)

**Fig. 4:**
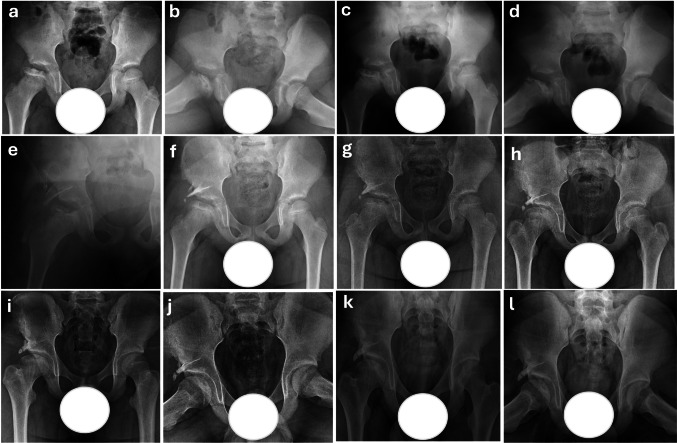
Serial radiographic follow-up of a 6-year-old male patient who presented at the fragmentation stage with Herring Group C classification and underwent shelf acetabuloplasty as a surgical containment procedure. Preoperative radiographs showing complete collapse of the lateral pillar, lateral subluxation of the femoral head, and multiple"head at risk"signs, including epiphyseal extrusion and metaphyseal changes (**a**, **b**). Preoperative 1-year follow-up radiographs displaying the reossification phase with significant lateral epiphyseal extrusion, despite ongoing remodeling, indicating persistent containment failure (**c**, **d**). Early postoperative radiograph displaying the immediate postoperative appearance following shelf acetabuloplasty (**e**). 1-year postoperative radiograph showing improved femoral head containment, although sphericity remains altered (**f**). 2-year postoperative radiograph demonstrating well-maintained containment and significant improvement in femoral head sphericity, indicating favorable remodeling (**g**). 3-year postoperative radiograph displaying continued remodeling with good femoral head sphericity (**h**). 5-year postoperative radiographs showing good joint congruity, with a fracture line visible at the distal end of the shelf graft, though no resorption is present (**i**, **j**). 7-year final follow-up radiographs demonstrating a well-shaped femoral head morphology with excellent joint congruity, classified as Stulberg Class I (**k**, **l**)

**Fig. 5:**
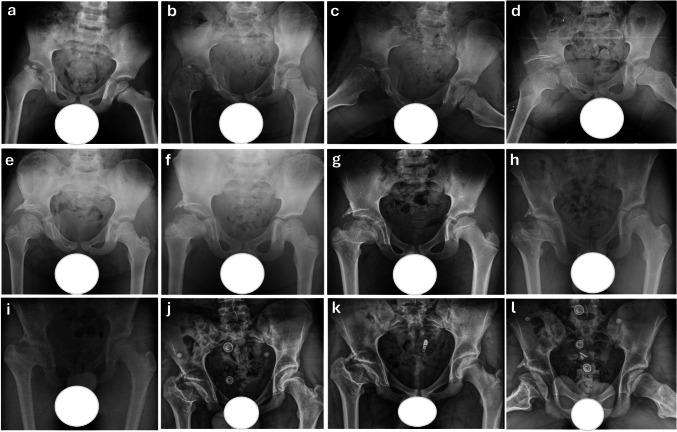
Serial radiographic follow-up of a 7-year-old male patient who initially presented at the fragmentation stage (Waldenström Grade 2) and was initially managed conservatively. Due to persistent femoral head containment failure, the patient subsequently underwent shelf acetabuloplasty. Preoperative radiograph showing Herring Group C classification with severe and extensive femoral head involvement. Significant lateral subluxation is present, along with pronounced epiphyseal extrusion (**a**). 1-year follow-up radiographs demonstrating ongoing reossification. Lateral subluxation of the femoral head remains prominent, indicating persistent containment failure (**b**, **c**). Early postoperative radiograph at age 9 following shelf acetabuloplasty (**d**). 1-year postoperative radiograph demonstrating graft fusion with good femoral head coverage and sphericity; however, lateral subluxation persists, and coxa magna is present (**e**). 2-year postoperative radiograph showing a spherical femoral head with maintained congruity. However, coxa magna is evident along with a shortened femoral neck (**f**). 4-year postoperative radiograph showing the initial loss of femoral head sphericity, though flattening has not yet occurred. The femoral head exhibits an ovoid, mushroom-like shape with associated coxa magna. Femoral neck shortening is also evident (**g**). 5-year postoperative radiograph showing early signs of femoral head remodeling. Although sphericity is lost, joint congruity remains preserved (**h**). 6- and 7-year postoperative radiographs demonstrating progressive improvement in femoral head morphology. At the seven-year follow-up, acetabular steepening becomes more prominent (**i**, **j**). 8-year postoperative final follow-up radiographs showing flattening of both the femoral head and acetabulum with aspherical congruency, classified as Stulberg Class IV (**k**, **l**)

Among the radiographic classifications, only the Herring Lateral Pillar Classification showed a statistically significant difference between groups (*p* = 0.001), with all Shelf group patients classified as Group C. Other classification-based outcomes, including Waldenström stage and Stulberg scores, were similar between groups (*p* > 0.05) (Table 2). Representative radiographic follow-ups of patients treated with conservative management are shown in Figs. 2 and 3, while those treated with shelf acetabuloplasty are demonstrated in Figs. 4 and 5, illustrating typical morphological outcomes.

**Table 2:** Comparison of radiographic classification systems between Shelf and Conservative Treatment Groups

Variables	Shelf Group (*n* = 14)		Conservative Group (*n* = 14)		Between-group comparison (*p-values)*
	Initial assessment	Final follow-up assessment	Initial assessment	Final follow-up assessment	
Waldenström Grade	Stage 2: 2 (14.3%) Stage 3: 9 (64.3%) Stage 4: 3 (21.4%)	-	Stage 1: 2 (14.3%) Stage 2: 3 (21.4%) Stage 3: 9 (64.3%)	-	0.218
Herring Lateral Pillar Classification	Group C: 14 (100%)	-	Group B: 6 (42.9%) Group B/C: 6 (42.9%) Group C: 1 (7.1%)	-	0.001*
Stulberg Classification		Class I: 2 (14.3%) Class II: 3 (21.4%) Class III: 5 (35.7%) Class IV: 4 (28.6%)		Class I: 4 (28.6%) Class II: 4 (28.6%) Class III: 3 (21.4%) Class IV: 2 (14.3%) Class V: 1 (7.1%)	0.637
Radiographic outcomes as per Modified Stulberg Classification)		Good: 5 (35.7%) Fair: 5 (35.7%) Poor: 4 (28.6%)		Good: 8 (57.1%) Fair: 3 (21.4%) Poor: 3 (21.4%)	0.623
Presence of'Head at Risk'Signs	Lateral Subluxation: 14 (100%)	-	Lateral Subluxation: 13 (92.9%)	-	1.000
	Gage Sign: 4 (28.6%)		Gage Sign: 4 (28.6%)		1.000
	Calcification: 9 (64.3%)		Calcification: 2 (14.3%)		0.009*
	Metaphyseal Changes: 11 (78.6%)		Metaphyseal Changes: 11 (78.6%)		0.339
	Horizontal Growth Plate: 9 (64.3%)		Horizontal Growth Plate: 8 (57.1%)		0.500

All values are presented as frequencies and percentages (n, %)

The statistical significance level was set at *p* < 0.05

Within-group comparisons were conducted using the Fisher-Freeman-Halton exact test

Statistically significant differences are denoted with an asterisk (*)

#### Quantitative radiographic measurements

At final follow-up, the Shelf group demonstrated significantly better radiographic parameters compared to the Conservative group, including higher center–edge angle and femoral head coverage ratio, and lower Sharp angle (*p* < 0.001 for all). Other quantitative measurements, including femoral neck shaft angle, did not show significant between-group differences **(**Table 3**).**

**Table 3:** Comparison of Radiographic Measurements Between Shelf Acetabuloplasy and Conservative Treatment Groups

*Variables*	Shelf Group (*n* = 14)		Within-group comparisons (*p-values)*	Conservative Group (*n* = 14)		Within-group comparisons *(p-values)*	Between-group comparison at initial assessment (*p-values)*	Between-group comparison at final follow-up (*p-values)*
	Initial assessment	Final assessment		(Initial)	(Final)			
Femoral Head Subluxation Ratio	1.90 ± 0.26 (1.50–2.33)	1.22 ± 0.16 (1.05–1.60)	< 0.001*	1.73 ± 0.49 (1.25–2.50)	1.51 ± 0.63 (1.00–3.00)	0.259	0.267	0.104
Femoral Head Size Ratio	1.32 ± 0.16 (1.02–1.60)	1.17 ± 0.10 (1.05–1.39)	0.003*	1.26 ± 0.11 (1.10–1.50)	1.20 ± 0.10 (1.05–1.50)	0.121	0.225	0.445
Wiberg Center–Edge Angle	19.71 ± 7.11 (8.00–34.00)	48.43 ± 5.53 (36–58)	< 0.001*	23.71 ± 9.40 (7–47)	28.93 ± 10.11 (14–49)	0.112	0.215	< 0.001*
Sharp Angle	44.79 ± 6.89 (37–64)	32.93 ± 4.57 (27–43)	< 0.001*	46.29 ± 3.81 (42–54)	42.86 ± 4.26 (35–49)	0.030*	0.646	< 0.001*
Femoral Head Coverage Ratio	63.86 ± 6.99 (53–75)	96 ± 8.83 (70–107)	< 0.001*	66.57 ± 8.87 (51–82)	73.57 ± 10.28 (58–90)	0.059	0.433	< 0.001*
Femoral Neck Shaft Angle	141 ± 5.56 (132–153)	132 ± 4.51 (120–138)	< 0.001*	138.43 ± 6.53 (127–153)	134.00 ± 8.85 (120–154)	0.053	0.272	0.458

All variables are presented as mean ± SD (min–max). The statistical significance level was set at *p* < 0.05

Within-group comparisons were conducted using the Paired-Samples T-Test

Between-group comparisons were performed using the Independent-Samples T-Test

Statistically significant variables are denoted with an asterisk (*)

Fusion was obtained in all shelf grafts. At the final follow-up, a fracture at the distal end of the graft was identified in a patient; however, it remained asymptomatic and did not result in resorption. Additionally, another patient exhibited graft resorption at the distal end during the follow-up period. No further graft-related complications were detected.

### Clinical outcomes

At the final follow-up, the HHS was significantly higher in the Shelf group (67.93 ± 15.99 (52–97)) than in the Conservative group (54.57 ± 13.30 (43–81), *p* = 0.024) (Table 4).

**Table 4:** Comparison of Harris Hip Score between Shelf and Conservative groups, including subgroup analyses based on the Stulberg classification

Variables mean ± SD (min–max)	Stulberg Class I-II *(good)*	Stulberg Class III *(Fair)*	Stulberg Class IV-V *(Poor)*	*P* values^a^	Overall group participants
Shelf Group					
Harris Hip Score	69.00 ± 10.65 (52–77)	68.80 ± 19.28 (52–97)	65.50 ± 21.21 (52–97)	0.946	67.93 ± 15.99 (52–97)
Conservative Group					
Harris Hip Score	54.38 ± 14.18 (43–81)	50.33 ± 3.51 (47–54)	59.33 ± 19.30 (44–81)	0.741	54.57 ± 13.30 (43–81)
					*p* = 0.024^b^

All variables are presented as mean ± SD (min–max)

The statistical significance level was set at *p* < 0.05

^a^One-way ANOVA was performed to evaluate statistical differences among the subgroups

^b^Student’s T test was performed for between-group comparison

### Isokinetic strength measurements

Compared to the Shelf and Control groups, the Conservative group exhibited significantly lower extension, abduction, and adduction peak torque values, as well as reduced work fatigue ratios in all tested muscle groups (*p* < 0.001 for all) (Table 5). No significant differences were found between the Shelf and Control groups in these parameters. Flexion-related strength and endurance values did not differ significantly among the three groups.

**Table 5:** Comparison of peak torque and work fatigue ratio values among the Shelf, Conservative, and Control groups

Variables *(Mean* ± *SD, min–max)*	Shelf Group (*n* = 14)	Conservative Group (*n* = 14)	Control Group (*n* = 14)	*p*-Values (ANOVA)	Post-Hoc results
Peak Torque Measurements *(Nm)*					
Flexion	61.50 ± 18.67 (20–89)	66.29 ± 17.60 (43–94)	61.43 ± 10.97 (42–85)	0.661	No significant difference (*p* = 0.714)^a^
Extension	109.43 ± 37.41 (31–176)	52.79 ± 29.95 (20–118)	108.29 ± 17.57 (83–149)	< *0.001*	**Shelf, Control > CT**^a^ (*p* < 0.001; Shelf ≈ Control, *p* = 0.994)
Abduction	48.86 ± 18.90 (18–91)	29.79 ± 21.59 (9–81)	60.57 ± 11.49 (45–85)	< *0.001*	**Control > Shelf > CT**^a^ (*p* < 0.001; Control ≈ Shelf, *p* = 0.205)
Adduction	79.21 ± 25.67 (38–133)	38.21 ± 33.44 (12–104)	72.07 ± 10.21 (60–94)	< *0.001*	**Control, Shelf > CT**^a^ (*p* < 0.001; Control ≈ Shelf, *p* = 0.733)
Work Fatigue Ratios *(%)*					
Flexion	82 ± 25.24 (27–115)	59.50 ± 34.91 (27–142)	94.36 ± 15.66 (65–120)	*0.004*	**Control > CT,** **Shelf ≈ Control, Shelf ≈ Conservative**^b^ (*p* < 0.001; Shelf vs. Control = 0.440; Shelf vs. Conservative = 0.075)
Extension	163.29 ± 53.43 (43–236)	62.43 ± 57.66 (16–179)	173.21 ± 27.09 (125–218)	< *0.001*	**Control, Shelf > CT**^a^ (*p* < 0.001; Control ≈ Shelf, *p* = 0.733)
Abduction	24.64 ± 9.46 (8–41)	19.21 ± 9.37 (8–45)	31.79 ± 7.76 (20–50)	*0.002*	**Control > Shelf > CT**^a^ (*p* < 0.001; Control ≈ Shelf, *p* = 0.205)
Adduction	44.21 ± 15.21 (20–75)	25.14 ± 15.38 (11–57)	41 ± 6.16 (33–54)	< *0.001*	**Control, Shelf > CT**^a^ (*p* < 0.001; Control ≈ Shelf, *p* = 0.733)

All variables are presented as mean ± SD (min–max)

*P*-values were obtained from ANOVA tests

The statistical significance level was set at *p* < 0.05

The selection of post-hoc tests was based on the results of Levene’s homogeneity test:

^a^Tukey HSD was used (Levene *p* > 0.05)

^b^Games-Howell was used (Levene *p* ≤ 0.05)

In the Shelf group, there were no statistically significant differences between the affected and unaffected sides in all tested muscle groups, while in the Conservative group, extension peak torque was significantly lower on the affected side compared to the unaffected side (*p* = 0.047) (Table 6).

**Table 6:** Comparison of peak torque and work fatigue ratios between affected and unaffected sides in shelf and conservative groups

Variables *(Mean* ± *SD, min–max)*	Shelf Group *(n* = *14)*		*P*-values	Conservative Group *(n* = *14)*		*P*-values
	Affected side	Unaffected side		Affected side	Unaffected side	
Peak Torque Measurements *(Nm)*						
Flexion	61.50 ± 18.67 (20–89)	60.43 ± 19.46 (22–92)	0.773	66.29 ± 17.60 (43–94)	67.71 ± 19.77 (43–106)	0.732
Extension	109.43 ± 37.41 (31–176)	114.64 ± 35.86 (45–191)	0.128	52.79 ± 29.95 (20–118)	60.64 ± 41.14 (20–141)	0.047
Abduction	48.86 ± 18.90 (18–91)	52.29 ± 20.43 (19–91)	0.262	29.79 ± 21.59 (9–81)	29.86 ± 20.46 (9–83)	0.962
Adduction	79.21 ± 25.67 (38–133)	77.36 ± 22.58 (35–117)	0.640	38.21 ± 33.44 (12–104)	42.50 ± 41.01 (12–121)	0.196
Work Fatigue Ratios *(%)*						
Flexion	82.00 ± 25.24 (27–115)	78.93 ± 25.18 (28–117)	0.483	59.50 ± 34.91 (27–142)	58.29 ± 29.71 (27–129)	0.853
Extension	163.29 ± 53.43 (43–236)	164.21 ± 50.63 (58–237)	0.887	62.43 ± 57.66 (16–179)	74.43 ± 73.53 (16–222)	0.078
Abduction	24.64 ± 9.46 (8–41)	26.07 ± 10.14 (11–47)	0.275	19.21 ± 9.37 (8–45)	19.21 ± 9.48 (8–47)	1.000
Adduction	44.21 ± 15.21 (20–75)	43.36 ± 13.31 (19–65)	0.683	25.14 ± 15.38 (11–57)	27.93 ± 20.30 (11–71)	0.154

All variables are presented as mean ± SD (min–max)

The statistical significance level was set at *p* < 0.05

## Discussion

This study has shown that shelf acetabuloplasty and conservative treatment could both provide comparable long-term femoral head morphology and hip joint congruity in children with LCPD, as assessed by the Stulberg classification. Nonetheless, better outcomes could be expected following shelf acetabuloplasty in HHS as well as some quantitative radiographic parameters. Specifically, the Wiberg centre–edge angle, femoral head coverage ratio, and Sharp angle exhibited significant improvements in the shelf group, implying that surgical containment may offer superior femoral head coverage. Given the lack of a significant difference in the Stulberg classification, these findings suggest that while shelf acetabuloplasty enhances femoral head coverage, its long-term effect on overall joint congruity is comparable to conservative treatment.

A major strength of the present study is the comparability of baseline characteristics between the shelf and conservative groups. At the initiation of treatment, both groups had similar Waldenström grades and comparable values in all quantitative radiographic parameters. However, an important difference was identified in the Herring lateral pillar classification, as all patients in the shelf group were classified as Herring Group C, although the conservative group predominantly involved Herring B or B/C cases. Despite this difference, the prevalence of ‘head at risk’ signs, including lateral subluxation, was comparable between the two groups, supporting the idea that both cohorts carried similar risk factors for developing femoral head deformity. This comparability is essential for interpreting the impact of surgical versus conservative treatment, particularly in patients with more advanced disease.

Our radiographic findings are consistent with previous studies reporting that shelf acetabuloplasty significantly improves key structural parameters such as the Wiberg centre–edge angle, Sharp angle, and femoral head coverage ratio in the long-term follow-up [6, 7, 18]. However, despite these improvements in acetabular coverage, its impact on long-term femoral head morphology remains unclear. To date, according to our review of literature, comparative studies evaluating the long-term radiographic outcomes of shelf acetabuloplasty and conservative treatment are extremely limited. The present study conducted this comparison to address a critical gap in understanding the long-term outcomes of patients undergoing shelf acetabuloplasty versus those managed conservatively. Considering the shelf acetabuloplasty can be performed as a salvage procedure in advanced-stage LCPD (Waldenström Stage III-IV, Herring Group C), an alternative approach for these children may be either continued conservative management or other surgical interventions. To objectively evaluate whether shelf acetabuloplasty could provide additional benefits over conservative treatment in this specific patient population, we selected a conservatively treated cohort that closely matched the preoperative characteristics of the shelf cohort.

To our knowledge, there exists only one investigation [19] that has performed a direct comparison between these two therapeutic approaches. Parmentier et al. [19] retrospectively reviewed 80 children (88 hips) treated for LCPD, with a mean follow-up of 6.3 years (range: 2.0 to 12.9 years), which is comparable to the long-term follow-up in our study. Among these, 47 hips underwent shelf acetabuloplasty, while 41 hips were treated nonoperatively. In agreement with our study’s results, they suggested that while the shelf procedure significantly improves acetabular coverage, it is not more effective than conservative treatment in preventing femoral head deformity based on the Stulberg classification. Although our study included fewer patients compared to Parmentier et al., the age- and gender-matched design minimizes potential bias, making our findings more directly comparable between treatment groups. In contrast, our results differ from those of Grzegorzewski et al. [20] (2013) and Santana et al. [21] (2024). They indicated that surgical containment methods, such as shelf acetabuloplasty, resulted in better radiographic outcomes, indicating a greater rate of Stulberg I-II classifications compared to conservative methods. These discrepancies may be attributed to differences in patient selection criteria, baseline disease severity, and the timing of surgical intervention. Additionally, variations in follow-up duration and radiographic assessment methodologies could contribute to these contrasting findings, highlighting the complexity of determining the optimal treatment approach for LCPD.

The present study has indicated significant differences in HHS and isokinetic hip muscle function between the treatment groups in the mid- to long-term follow-up of patients with LCPD. Previous studies examining the outcomes of the shelf acetabuloplasty primarily focused on pain relief and radiographic findings but did not evaluate isokinetic muscle strength and endurance [9, 18, 22]. To our knowledge, this is the first study to assess isokinetic hip muscle function following shelf acetabuloplasty and conservative treatment using an age- and gender-matched study design, including a healthy control cohort for direct comparison. This approach provides a unique opportunity to quantify mid-to long-term muscle performance deficits associated with different treatment strategies in LCPD. Isokinetic assessments shed light on two important facets of muscular performance: peak muscle strength, reflecting the maximum torque a muscle can generate, and work fatigue ratio, representing the muscle's capacity to perform repeated contractions without excessive decline in force output. These two parameters are crucial in evaluating both dynamic stability and endurance, which are critical for activities of daily living and long-term joint function.

In terms of peak muscle strength, our results showed that while hip flexion and extension torque values in the shelf group were comparable to those in the control group, hip abduction and adduction torque values were significantly lower than in healthy individuals. This suggests that while shelf acetabuloplasty may help preserve hip flexor and extensor muscles, it does not fully restore the strength of hip abductors and adductors to normal levels. Notably, the conservative treatment group exhibited significantly lower peak torque values in hip extension, abduction, and adduction compared to both the shelf and control groups, suggesting that nonoperative management may be associated with greater long-term muscle strength deficits.

Concerning the work fatigue ratio, which is a measure of muscular endurance, the children who received conservative treatment exhibited significantly lower values in hip extension, abduction, and adduction. This suggests that they were more susceptible to muscle fatigue. Additionally, the shelf and control groups did not demonstrate any substantial differences in fatigue metrics, which supports the notion that surgical containment may more effectively maintain muscular endurance over time than conservative management. These results indicate that reduced muscle endurance following the conservative treatment may have a detrimental effect on long-term functional outcomes, potentially leading to gait disturbances, early fatigue, and reduced joint stability. Overall, restoring hip joint integrity in LCPD is not only about obtaining radiographic perfection but also about optimizing long-term function and mobility. Although Stulberg radiographic outcomes were comparable between shelf and conservative groups, patients who underwent surgical containment exhibited significantly higher HHS along with better muscle strength and endurance. These findings could be interpreted as that the advantages of shelf acetabuloplasty extend beyond femoral head remodeling, potentially improving functional mobility and reducing long-term musculoskeletal impairment. Further research with larger cohorts and dynamic gait analyses is needed to better understand the clinical implications of these observations.

Although shelf acetabuloplasty has recently been employed in the early stages of LCPD to promote femoral head remodeling [8], our study adhered to a more traditional approach [6, 7], implementing it as a salvage procedure in cases with lateral subluxation and epiphyseal extrusion. By reinforcing acetabular coverage, the procedure aimed to enhance femoral head containment and prevent progressive joint deterioration in these high-risk patients. In this salvage setting, our results demonstrate the efficacy of shelf acetabuloplasty, as reflected by preservation of isokinetic hip muscle function and favourable mid- to long-term radiographic outcomes.

This study has several limitations. First, the small sample size may have limited the statistical power, and future multi-center studies with larger cohorts are needed to validate our findings. Second, its retrospective design introduces potential selection bias, despite efforts to match the conservative treatment group with the shelf group based on preoperative characteristics. Lastly, while our follow-up was sufficient for radiographic and isokinetic muscle assessments, longer-term studies are needed to determine whether these findings impact osteoarthritis progression and long-term hip survival.

## Conclusion

This study suggests that shelf acetabuloplasty may be an effective salvage procedure for advanced-stage LCPD with lateral subluxation in improving femoral head containment. Comparable mid- to long-term radiographic outcomes can be obtained by both shelf acetabuloplasty and conservative management, with most patients achieving good to fair Stulberg results. In addition to radiographic findings, our results suggest that shelf acetabuloplasty may offer potential advantages in clinical outcomes and isokinetic hip muscle performance. These preliminary results highlight the importance of incorporating objective functional assessments in evaluating treatment efficacy. Following Shelf acetabuloplasty, comparable hip flexor and extensor strength to healthy controls can be expected, although mild abductor and adductor weakness may persist. Therefore, targeted rehabilitation strategies focusing on hip abductor and adductor strength may further improve post-operative recovery.

## Data Availability

No datasets were generated or analysed during the current study.
